# The Role of Botulinum Toxin for Masseter Muscle Hypertrophy: A Comprehensive Review

**DOI:** 10.3390/toxins17020091

**Published:** 2025-02-14

**Authors:** Martina Ferrillo, Eleonora Sommadossi, Loredana Raciti, Dario Calafiore, Kamal Mezian, Valeria Tarantino, Michele Vecchio, Umile Giuseppe Longo, Luigi Losco, Alessandro de Sire

**Affiliations:** 1 Dentistry Unit, Department of Health Sciences, University of Catanzaro “Magna Graecia”, 88100 Catanzaro, Italy; 2 Private Practice, 10121 Turin, Italy; sommadossi.e@gmail.com; 3 Unità Spinale Unipolare, Azienda Ospedaliera per le Emergenze Cannizzaro, 98102 Catania, Italy; loredana.raciti79@gmail.com; 4 Physical Medicine and Rehabilitation Unit, Department of Neurosciences, ASST Carlo Poma, 46100 Mantova, Italy; dario.calafiore@asst-mantova.it; 5 Department of Rehabilitation Medicine, First Faculty of Medicine, Charles University and General University Hospital in Prague, 12800 Prague, Czech Republic; kamal.mezian@lf1.cuni.cz; 6 Private Practice, 90142 Palermo, Italy; tarantinov4@gmail.com; 7 Department of Biomedical and Biotechnological Sciences, University of Catania, 95123 Catania, Italy; michele.vecchio@unict.it; 8 Rehabilitation Unit, “AOU Policlinico G. Rodolico-San Marco”, 95123 Catania, Italy; 9 Fondazione Policlinico Universitario Campus Bio-Medico, Via Alvaro del Portillo 200, 00128 Roma, Italy; g.longo@policlinicocampus.it; 10 Research Unit of Orthopaedic and Trauma Surgery, Department of Medicine and Surgery, Università Campus Bio-Medico di Roma, Via Alvaro del Portillo 21, 00128 Roma, Italy; 11 Department of Medicine, Surgery and Dentistry, University of Salerno, 84081 Baronissi, Salerno, Italy; llosco@unisa.it; 12 Physical Medicine and Rehabilitation, Department of Medical and Surgical Sciences, University of Catanzaro “Magna Graecia”, 88100 Catanzaro, Italy; alessandro.desire@unicz.it; 13 Research Center on Musculoskeletal Health, MusculoSkeletalHealth@UMG, University of Catanzaro “Magna Graecia”, 88100 Catanzaro, Italy; 14 Department of Rehabilitation and Sports Medicine, Second Faculty of Medicine, Charles University and University Hospital Motol, 15006 Prague, Czech Republic

**Keywords:** masseter muscle hypertrophy, lower face contouring, botulinum toxin type A

## Abstract

Masticatory muscle hypertrophy (MMH) is a rare clinical phenomenon of uncertain etiology, characterized by a soft swelling near the angle of the jaw. This abnormal enlargement of the masseter muscle can alter the facial profile, leading to aesthetic concerns. Moreover, MMH may also have significant functional repercussions, including pain in the masseter region, often associated with temporomandibular disorders, fatigue, and discomfort during mastication. Non-conservative approaches offer an effective and minimally invasive solution by inducing localized muscle relaxation and reducing hypertrophy. Botulinum neurotoxin type A (BoNT/A) represents a therapeutic option for managing MMH, considering that injections can effectively reduce the masseter muscle volume, improving both facial aesthetics and related symptoms. Currently, the standard non-surgical management of MMH is BoNT/A injections, although consensus on the average dosage has not been definitely reached; on the other hand, there are data available in the literature about the injection technique of BoNT/A for lower face contouring. Therefore, the present comprehensive review aimed at exploring in detail the role of BoNT/A in the treatment of masseter muscle hypertrophy, describing its mechanism of action, the administration protocols, the clinical effects, and any side effects.

## 1. Introduction

The masseter muscle is one of the primary muscles of mastication and plays a crucial role in mandibular movement and masticatory function. However, under certain circumstances, there can be an uncommon condition characterized by an abnormal increase in its size, defined as “masticatory muscle hypertrophy” (MMH) [1]. MMH has a high incidence in the second and third decades of life, with no sex predilection, can occur unilaterally or more commonly bilaterally (in about 60% of cases) [2,3], and although the condition is generally benign, can significantly impact both aesthetics and function [4]. Functional problems may manifest as protrusion of the jaw, pain, or headache [5]. The exact etiology remains unclear; however, it is most regarded as an adaptive response to chronic functional stimuli or repeated muscle stress [5]. Contributing factors such as masticatory habits—including excessive gum chewing, clenching, and bruxism—or genetic predispositions have been reported [6]. When no specific etiological factors can be identified, an idiopathic origin should be considered (IMMH) [7]. Notably, certain conditions such as psychological disorders or emotional disturbances for developing IMMH have been identified; these risk factors alter proprioception and affect the ability to maintain proper tone in the masseter muscle [2].

From an aesthetic perspective, MMH can significantly alter the facial profile by enlargement of the masseter and/or temporalis muscles, often leading to functional and aesthetic concerns. The increase in muscle volume can give the mandible a squared and angular appearance, affecting the perception of facial symmetry and harmony. This aesthetic effect often concerns patients, prompting them to seek treatments to improve their facial appearance [8]. MMH can have significant functional repercussions. Patients may report localized pain in the masseter region, often associated with temporomandibular disorders (TMDs). Patients may also experience fatigue or discomfort during mastication due to increased muscle tension and altered biomechanics [2].

This condition can impair masticatory efficiency and contribute to worsening TMDs, further exacerbating discomfort or chronic muscle tension [9,10,11]. In some cases, excessive muscle activity can impair masticatory function, causing difficulty in mandibular movements and contributing to bruxism [4]. Diagnosis of unilateral MMH could be made based on clinical examination and radiological findings [2]. The treatment of MMH varies depending on the severity of the condition and the patient’s needs. Conservative treatments—such as the use of occlusal splints or night guards, oral devices to reduce muscle activity during sleep, physiotherapy as muscle massages and relaxation techniques to alleviate muscle tension, and behavior modification—aim to alleviate symptoms by reducing muscle hyperactivity and stress such as teeth grinding and excessive chewing of gum [6,12,13]. Non-conservative approaches, including botulinum toxin injections, offer an effective and minimally invasive solution by inducing localized muscle relaxation and reducing hypertrophy [14]. More in detail, botulinum neurotoxin type A (BoNT/A) represents a known therapeutic option for managing MMH considering that injections can effectively reduce the masseter muscle volume, improving both facial aesthetics and related symptoms [6,8,14,15]. The effect duration is temporary, requiring repeated treatments, but it offers a minimally invasive solution with a low risk of complications. Botulinum toxin type A (BoNT/A) injections are currently the standard non-surgical treatment for masseter muscle hypertrophy (MMH); however, a definitive consensus on the average dosage has yet to be established. Nevertheless, the literature provides substantial data on BoNT/A injection techniques for lower face contouring [14,16,17].

Therefore, the present comprehensive review aimed at exploring in detail the role of BoNT/A in the treatment of masseter muscle hypertrophy, describing its mechanism of action, the administration protocols, the clinical effects, and any side effects.

## 2. Methods

Articles were selected through a comprehensive literature search in databases such as PubMed, Scopus, and Web of Science. The search strategy included keywords such as “botulinum toxin”, “masseter muscle hypertrophy”, “mechanism of action”, “side effects”, and “treatment outcomes.” Inclusion criteria focused on studies published in peer-reviewed journals, primarily in English, and addressing the clinical applications, mechanism of action, or side effects of the botulinum toxin in masseter hypertrophy. Exclusion criteria included case reports and studies with insufficient data.

## 3. Masseter Muscle Hypertrophy

MMH is a rare clinical phenomenon of uncertain etiology [18], characterized by a soft swelling near the angle of the jaw, which may be associated with facial pain, significantly impacting facial aesthetics and functionality [19]. It can affect one side or both sides of the face and is characterized by an increase in the volume of muscle tissue, resulting in a change in the contour of the face [20]. In the literature, MMH is often described as a condition concomitant with the hypertrophy of other masticatory muscles, such as the temporalis muscles [21] or even medial pterygoid muscles [22]. Bilateral widening of the masseter muscles is more common but constitutes a minor aesthetic problem while maintaining facial symmetry [21]. Although the exact etiology of acquired hypertrophy of the masseter muscle is not well-known, there are several factors associated with its development, including the following: bruxism, TMD, pain, emotional stress, and oral parafunctions, including excessive unilateral chewing [6,22,23,24]. A recent study has demonstrated that intensive gum chewing can increase the stiffness of the masseter muscle and lead to bilateral hypertrophy of the masseter [25]. On the other hand, findings from several researchers suggest that the increase in muscle size is not caused by work-related hypertrophy but is the result of compensatory enlargement due to the lack of a specific type of muscle fiber [26]. Furthermore, along with the increase in muscle mass, changes may occur in the adjacent bone tissue, such as a thickening of the cortex of the angle and ramus of the mandible, the temporal fossa, and the zygomatic arch with a corresponding decrease in the area of the medulla or prominent exostoses at the angle of the mandibular bone, visible on computed tomography (CT) scans [27]. Bilateral enlargement of the masseter muscles is often accompanied by pain, which may be intermittent and may be confused with pain arising from the parotid gland [28,29]. Clinical examination usually reveals a mass of soft tissue near to the angle of the jaw, which becomes more prominent when clenching the teeth [30]. A limitation of mouth opening has been reported in some cases, particularly when the muscles are focally dystonic with tension in the region of the hypertrophic muscle [31]. It should be noted that the composition of muscle fibers in the enlarged masseter is very different from similar “work hypertrophy” muscles and healthy masseter muscles [32], suggesting that the term “hypertrophy” may be potentially misleading. Most patients report a slow but progressive nature of the condition [22,27]. The pathophysiology of muscle tone in patients with masseter muscle hypertrophy has not been studied. The increase in masticatory muscle tone is related to the increase in masticatory forces, generated by the masticatory muscles [14]. Both parameters increase negatively during parafunction, which means harmful movement habits (such as grinding, clenching, or chattering teeth, biting nails, pressing the tongue against the teeth, and frequently chewing gum) that do not relate to physiological activities [33]. Stal et al. [34] pointed out that there is a different reactivity of masticatory muscles and skeletal muscles to stress because both these muscle groups have different embryonic origins; the masticatory muscles come from the mesenchyme of the first pharyngeal arch, while the skeletal muscles come from the mesoderm of the somite called the myotome, and Korfage et al. [35] highlighted the important advantage of diversity of fiber types, such as that observed in jaw muscles. Indeed, compared to the muscles of the limbs and trunk, the masticatory muscles possess a higher percentage of hybrid fibers, which express many subtypes of myosin or a greater number of type I fibers [36]. A large amount of hybrid fibers is associated with the plasticity of masticatory muscles and more efficient energy consumption during contraction [37]. Compared to the masticatory muscles, limb and trunk muscles have type II fibers with a larger diameter than type I. This suggests that the high presence of type I fibers with a small cross-sectional area (CSA) in masticatory muscles could facilitate the greater exchange of nutrients and O_2_ with the extracellular environment, increasing the fibers’ resistance to fatigue [38]. Excessive and prolonged muscle tension becomes the cause of non-physiological and excessive overload of the TMJ, which in turn leads to damage to the soft tissue elements inside the joints [39].

## 4. Botulinum Toxin: Mechanisms of Action and Indications

Botulinum toxin is a neurotoxin produced by the anaerobic bacterium *Clostridium botulinum*, consisting of a neurotoxic core and non-toxic proteins. There are seven serotypes of BT (A, B, C, D, E, F, and G), each of which has additional subtypes (e.g., subtype A contains several distinct subtypes). The growing use of botulinum neurotoxins (BoNTs) in medical and aesthetic applications has spurred the development of various BoNT products [40]. All serotypes have a similar chemical structure and are neurotoxins, except for subtype C2, which belongs to the family of binary AB-type toxins, structurally composed of separate enzymatic (A, C2I) and binding (B, C2II) components [41]. 

Among its seven serotypes, the BoNT/A is the most extensively studied and clinically applied, particularly in aesthetic medicine, oncology, neurology, rehabilitation, and dentistry [42,43,44,45]. Differences among the botulinum toxin serotypes lie in their specific protein targets and clinical applications. For instance, BoNT/A and BoNT/B target distinct proteins in the SNARE complex; while BoNT/A cleaves the SNAP-25 protein, BoNT/B specifically targets synaptobrevin (VAMP), another critical component of the vesicle fusion machinery. This distinction contributes to their unique pharmacological profiles. BoNT/A is the primary choice for aesthetic and neurological applications due to its well-characterized safety and efficacy, whereas BoNT/B is sometimes preferred in cases of resistance to BoNT/A, such as in cervical dystonia, where its alternative protein target may overcome immunogenicity issues [45,46,47].

Upon injection, BoNT/A is endocytosed into the cell and cleaves the C-terminal portion of SNAP-25. This cleavage disrupts the SNARE complex, blocking the exocytosis of neurotransmitters and the acetylcholine release [48]. Additionally, BoNT/A suppresses the peripheral release of neuropeptides and inflammatory mediators, including CGRP (calcitonin gene-related peptide), which plays a key role in pain signaling and inflammation [49,50,51]. The reduction in CGRP in the peripheral and central nervous system is thought to contribute to its therapeutic effects in migraine management [41,52,53]. CGRP release occurs in the meninges and the trigeminal caudal nucleus and is amplified by P2Y purinergic receptors on satellite glial cells [54], which are influenced by factors such as 17-beta-estradiol. Conversely, P2X3 receptor-mediated peripheral pain signaling in neuronal afferents can attenuate CGRP activity [55]. Furthermore, receptors such as the transient receptor potential vanilloid 1 (TRPV1) play a critical role in pain perception and sensitization. These receptors may represent additional therapeutic targets for pain management [56]. The toxin consists of a 100 kDa heavy chain (HC) and a 50 kDa light chain (LC) linked by a disulfide bond. The heavy chain facilitates receptor binding and endocytosis into the neuron, while the light chain cleaves SNAP-25—a component of the SNARE complex—to inhibit acetylcholine release at the neuromuscular junction. This process leads to a reversible flaccid paralysis, a key therapeutic mechanism in conditions like spasticity, dystonia, and migraine.

BoNT/A has demonstrated significant effects in treating MMH, bruxism, TMDs, and oromandibular dystonia, especially in terms of function recovery [57]. Indeed, intra-muscular injections into the masseter and anterior temporalis muscles reduce muscle tone and bulk, alleviating pain and improving jaw functionality. The therapeutic effect of intramuscular injections depends on the section of the muscle, the dose administered, and the injection method [58,59]. Electromyographic studies confirm the decreased neuromuscular activity post-injection, with effects lasting 3–4 months [49,59]. Additionally, the reduced formation of axonal bundles during recovery promotes sustained muscle relaxation, enhancing therapeutic outcomes [60]. In neurology, BoNT/A has become a cornerstone therapy for chronic migraine. By targeting CGRP release in the trigeminal system and modulating pain transmission pathways, the toxin offers a dual benefit—reducing nociceptive signaling and peripheral sensitization. Emerging studies also suggest that receptors like TRPV1 and purinergic P2X3 may represent complementary targets in pain management, further broadening the therapeutic potential of BoNT-A [52,56].

## 5. How to Treat a “Square Face”: Role of Botulinum Toxin

BoNT/A is the most popular subtype for injection for aesthetic purposes. To date, botulinum toxin has been widely and mainly used for the treatment of conditions affecting the upper and middle face; however, due to recent efforts and patients’ needs, the indications for cosmetic reasons of botulinum toxin injection have been expanded to the lower face and neck areas [59]. The use of botulinum toxin for the aesthetic contouring of the lower face is extending, and it is typically achieved by injecting the masseter muscle.

The most common cosmetic complaint for which patients seek treatment is a ”square face”, and the solution is the injection of botulinum toxin into the abovementioned muscle to decrease muscle hypertrophy. A triangular-shaped lower part of the face with a perfect jawline is the most desired shape by women compared to a wide and square one, which for many is considered more attractive in males. The aesthetics of the lower third of the face is determined by three main factors: size of the bony jaw, mass of the masseter muscle, and volume of the subcutaneous adipose tissue [60]. The size and shape of the jaw are predetermined at birth and are fixed at adulthood; these factors also vary between different ethnicities and races. The volume of subcutaneous fat varies from person to person based on their individual body mass index and can change over time, according to general health and body fat percentage [60]. Masseter hypertrophy can result in a wide jaw, which negatively affects the aesthetic appearance of patients [61]. The procedure is performed by injection with a 30 G needle inserted deep into the muscle; the injection safety zone can be easily determined. The inferior border of the mandible is marked, which is the lower margin of the injection zone; the upper margin is a line drawn from the corner of the mouth to the earlobe. The patients are asked to grit the teeth so that the anterior and posterior borders of the masseter muscle can be palpated and marked. Thus, a rectangle shape is drawn on the lower face, and the injection safety zone is depicted within this rectangle, 1 cm from the margins [61]. Operating within this area, recommendations most commonly suggest three injection points, using a total amount of 24 to 60 U of onabotulinumtoxinA or incobotulinumtoxinA and 60 to 300 U of abobotulinumtoxinA [60,61]. Chang et al. [62] have reported a favorable long-term efficacy for this method, with a 12% reduction in masseteric muscle volume after three consecutive injections, 1 year after completion of the treatment. The minimum dosage used in a previous study for toxin injection into the masseters was 20 U of onabotulinumtoxinA on each side with two injection sessions at a 4-month interval [63]. The standard non-surgical treatment for MMH currently involves BoNT/A injections; however, there is no definitive consensus on the average dosage. Nevertheless, the existing literature provides valuable insights into the injection techniques of BoNT/A for achieving lower face contouring [64,65,66].

While some authors argue that a multi-point injection allows for better distribution of the toxin into the muscle, single-point injections are quicker to perform and less painful, and may be less prone to producing complications [67]. In contrast, in 2018, Nikolis et al. [68] evaluated the effectiveness of two injection techniques in the treatment of 30 patients with MMH using the toxin incobotulinumtoxinA. Fifteen patients received 40U of incobotulinumtoxinA at a single injection point and fifteen patients received 40U with a 5-injection technique (8U for each injection). At the 6-month follow-up, they demonstrated no significant difference between the two injection techniques. It has been proven that botulinum toxin is a safe treatment, even after repeated sessions for several years. However, in most cases, there is a need for using a combination of botulinum toxin injection with other procedures to obtain optimal results [66,67]. An example of combination therapy would be fat grafting/autologous fat grafting with botulinum toxin injection into the masseters as a safe and satisfactory method for short and wide faces in order to normalize the ratios of different areas of the lower face and obtain a desirable oval face [69].

## 6. Role of Botulinum Toxin for Masseter Muscle Hypertrophy

Recently, the scientific literature has revealed a higher interest regarding the effectiveness and safety of BoNT/A, highlighting its potential as a non-invasive therapeutic option in masseter muscle hypertrophy [70]. A recent review performed by Kundu et al. [60] analyzed the effect of BoNT/A in masseter muscle, stating that botulinum toxin was consistently effective in relieving pain at 3 and 6 months after treatment with an effect that is probably dose-dependent. BoNT/A was consistently effective in relieving pain 3 and 6 months after treatment [59,60]. It is well-demonstrated that for each unit of increase in the dose of BoNT/A, the severity of masseteric hypertrophy decreased in terms of pain [62,69,70]. A recent study has demonstrated that a single injection in masseter muscle seems to have a clinically subjective and objective effect on masseteric hypertrophy [69,70]. Furthermore, patients who received three injections experienced a higher efficacy and longer duration of treatment maintenance compared with those who received two injections [71]. Several techniques of injecting BoNT/A into masseter muscle have been employed in the past. Some authors are used to the multi-point injection technique since it allows for a better distribution of the toxin in the muscle, while some use the lowest injection sites possible or a single injection site [69]. Moreover, several studies demonstrated the efficacy of a biphasic injection method that is associated with minimum self-resolving side effects [72,73,74,75]. The authors proved the role of extensive knowledge of muscular anatomy and an appropriate injection technique as key factors in desirable clinical outcomes and decreasing side effects [75]. Nevertheless, comparing the two methods of multi-injection and single-injection treatments, no superiority of either method was found. Moreover, it has been suggested that the analgesic effect of BoNT/A could involve the reduction in neurotransmitter release at both peripheral and central levels [73]. Consequently, BoNT/A may have a direct analgesic effect by blocking the transmission of pain signals from the temporomandibular area to the nervous system [74]. This might be due to BoNT/A’s ability to inhibit the release of certain neurotransmitters involved in pain signaling [60].

From a rehabilitative perspective, a recent systematic review of 2024 affirms that BoNT/A can improve the masseter hypertrophy and hypertonicity, significantly alleviating joint stress and improving jaw function [68]. Furthermore, several RCTs support its use for treating TMDs that can arise as a consequence of bruxism or independently [75,76]. The long duration of action of BoNT/A for pain relief, which can persist up to 6 months after treatment, could be due to the ability of its protease to avoid cellular degradation mechanisms and survive in the cell cytoplasm for a prolonged period [60]. In fact, it has been hypothesized that the pain-relieving effect of BoNT/A persists even after the return of muscle strength [77]. A potential explanation could also be that bruxists became more aware of parafunctional (teeth clenching) habits and took corrective measures, which may have restored the normal function of other masticatory muscles [78]. In this context, muscle thickness of the masseter muscle can play a pivotal role in orofacial pain. The use of BoNT/A can decrease the muscle’s thickness by an average of 31% after a 3-month follow-up, as measured by ultrasound, and volume loss continues even after restoration of the muscle function [67,79,80,81]. Recent studies showed a decrease in muscle thickness and an improvement in masseter contraction ability and masticatory performance. The authors conclude that a single injection of BoNT/A does not seem to affect muscle thickness—assessed by ultrasound—permanently since the thickness had recovered after 3 months [69]. However, the muscle’s thickness following a booster injection 3 months after the first did not return to normal after 6 months [69]. Previous animal studies and some human studies indicate that this could be a result of incomplete re-innervation of the injected area, fatty infiltration, fibrosis, and even atrophy due to necrosis of the muscle fibers in mice and humans [82]. It is interesting to note that the evaluation of muscle thickness using a caliper has shown that muscle thickness is directly proportional to the symptoms reported by the patient: after the injection, the thickness of the masseter decreases and, similarly, the symptoms disappear or are reduced [67]. Moreover, facial aesthetics also improve thanks to the reduction in thickness and muscle relaxation [83]. It is important to note that muscle thickness is often correlated with muscle strength. Thus, clinicians normally make dose adjustments based on the hypertrophy of the masseter muscle while avoiding the prevalence of side effects [84]. Moreover, it has been stated that the human system produces antibodies when exposed to excessive BoNT/A, which may impair its effectiveness in future treatment but, to date, BoNT/A has shown a very low protein content, avoiding this limiting effect [85,86,87]. To achieve personalized dose injections based on the ultrasound evaluation of masseter muscle and deep tendon thickness, Shi et al. [88] assessed the morphological changes in the masseter muscle and deep tendon of 206 subjects under relaxed and clenched conditions using ultrasound. The study found that the deep belly was thicker in both relaxed and clenched states in longitudinal ultrasound images; however, when Li et al. [18] used the same measurement method on 42 participants, they reached an opposite conclusion. Regarding muscle activity, compared to placebo injections, BoNT/A was associated with a significant decrease in occlusal force (kg) 3 months after injection, with a decrease in effects after 3 months and no significant differences at 6 months [89,90,91,92]. Comparing BoNT/A treatment with the use of occlusal bites and placebo injections, the analysis of maximum chewing force after BoNT/A injections demonstrated a significant reduction at 1 month or less. BoNT/A continued to outperform occlusal splints and saline placebo at 3 months [70]. Between 3 and 6 months, a significantly higher maximum chewing force was observed in the BoNT/A group compared to the oral splinting group [70]. No significant differences in maximal chewing force were observed between the BoNT/A and saline placebo groups [70]. BoNT/A works by controlling the intense contractions of the masticatory muscles [93]. Injection of the masseter alone is, therefore, sufficient to treat bruxism [94,95], with the other masticatory muscles (temporalis, pterygoids, digastrics, and genius-hyoids) capable of compensating and, therefore, supporting effective chewing [96]. The favorable mechanism of neuromuscular conduction block significantly reduces the tone of the masseter muscle, as confirmed by electromyographic (EMG) evaluation [97,98]. Furthermore, the recovery of conductivity occurs based on the formation of new axonal bundles, which are in much smaller quantities than the original connections, a very favorable phenomenon for achieving muscle relaxation [99]. In one study, post-injection polysomnographic evaluation confirmed that botulinum reduces the intensity of contraction of the masseter muscle for at least 12 weeks but does not modify the frequency of occurrence of rhythmic masticatory muscle activity (RMMA) episodes. On the other hand, the author offers a different perspective of bruxism: RMMA episodes are physiological so it is not necessary to intervene to try to reduce them and improve symptoms [89,98]. The efficacy of botulinum toxin treatment on the range of joint movements was observed: compared to a placebo, BoNT/A was associated with a significant increase in maximum pain-free opening (mm) 1 month after injection, and in unassisted opening (mm) at 1 month and 6 months post-injection; no difference was observed in maximum unassisted opening (mm) at 1 week or 2 months post-injection [67]. Compared to the placebo, BoNT/A was associated with a significant increase in the right lateral excursion (mm) at 1 week, 1 month, and 6 months; and in the left lateral excursion (mm) at 1 week, 1 month, and 6 months [67]. However, according to Asutay et al. [99], changes in voluntary mouth opening did not show statistically significant differences, unlike what was studied by Sidebottom et al. [100] and Guarda-Nardini et al. [101]. Other studies agree on an improvement in temporomandibular muscle and joint function in patients with muscular TMD [102,103,104,105,106,107,108]. In fact, compared to placebo injections, BoNT/A is associated with a significant post-injection reduction in rest pain intensity at 1 month, 2 months, 3 months, and 6 months from the first injection [102,103,104]. Additionally, BoNT/A was found to be associated with a significant decrease in masseter muscle activity at 1 month and occlusal force at 3 months [90]. Furthermore, BoNT/A resulted in a significantly greater increase in maximum pain-free mouth opening, unassisted mouth opening, and mandibular excursive movement at 1 month post-treatment [102]. Furthermore, several systematic reviews assessed the use of BoNT/A therapy for myofascial pain, reporting a reduction in pain intensity in the BoNT/A groups and concluding that this treatment was slightly more effective than placebo for pain reduction but appears as effective as other conservative and rehabilitative approaches [102,103,104,105]. Thus, some authors conclude that no strong recommendations can be drawn [106]. The therapeutic effect of intramuscular injections depends on the section of the muscle, the dose administered, and the injection method but the literature agrees on the duration of effects ranging from 3 to 4 months [59,72]. In summary, injection of a low dose of BoNT/A seems to have a clinically subjective and objective effect on masseteric hypertrophy. However, as a clinician, one must consider the possible harms that can occur with repeated injections in shorter intervals, which include impaired masticatory performance and muscle activity but also a permanent decrease in muscle thickness with consequent functional loss [107]. In comparison, the effects of BoNT/A generally last 3–6 months for aesthetic facial lines [108]. The longer duration of effectiveness after treatment of the masseters compared to treatment of the facial lines may be related to dosage [109].

## 7. Protocols of Botulinum Toxin Injections for Masseter Muscle Hypertrophy

Currently, the standard non-surgical management of MMH is BoNT/A injections, although a consensus on the average dosage has not been definitely reached; on the other hand, there are data available in the literature about the injection technique of BoNT-A for lower face contouring [14,16,17].

While some authors suppose that an injection in multiple injection sites allows for a better distribution of the toxin in the muscle and ensures a uniform reduction in the size of the masseter [102], single-point injections are quicker to perform and less painful, and may be less prone to producing complications. The data, therefore, suggest using as few injection sites as possible until more robust evidence is presented [110]. The most important concept when injecting BoNT/A into the masseter is to stay well within the confines of the muscle to avoid complications [62]. The ideal injection site is the most prominent point of the swollen masseter muscle, where the appropriate nerve innervates the affected muscle [66,111]. The number of BoNT/A injection sites and dosages may depend on the thickness of the masseter muscle and the degree of hypertrophy [66]. The superior portion of the masseter muscle is considered unsuitable for BoNT/A injection due to the location of the parotid duct and the absence of branches of the perforating nerve supplying the superficial layer of the masseter muscle [111]. To ensure proper targeting, clinicians can ask patients to clench their teeth, which facilitates the identification of the hypertrophic muscle [108]. A safe zone of the muscle lies below the line connecting the earlobe and the corner of the mouth, and between the posterior margin of the muscle and 1 cm after the corner of the mouth. Within this zone, most recommendations suggest using three injection points with a total dose of 24–60 units of onabotulinumtoxinA or incobotulinumtoxinA, or 60–300 units of abobotulinumtoxinA, delivered via a 30-gauge needle at appropriate depths into the muscle [110]. Li et al. [102] analyzed injection sites and the results indicated that at 6 months post-injection, BoNT/A injections to the masseter, temporalis, and pterygoid muscles were associated with greater pain reduction, while injections to the masseter muscles alone or masseter and temporalis muscles produced a smaller effect size. This suggests that broader targeting (masseter, temporalis, and pterygoid muscles vs. masseter and temporalis) may provide superior outcomes for pain management [102]. Despite the variability in dosing protocols, most studies agree that doses lower than 20 units are inadequate for significant results, while higher doses (20–40 units) yield better effects, particularly in patients with severe symptoms. The U.S. Food and Drug Administration (FDA) has approved a maximum dose of 400 units of onabotulinumtoxinA or incobotulinumtoxinA within a three-month interval. However, doses up to 600 units may not significantly increase the risk of adverse events. The neurotoxin load (in ng per 100U) varies among BoNT-A formulations: 0.73 ng/100U for onabotulinumtoxinA; 0.65 ng/100U for abobotulinumtoxinA; and 0.44 ng/100U for incobotulinumtoxinA. Consequently, the specific potency of the 150 kDa BoNT-A neurotoxin is estimated at 137 units/ng for onabotulinumtoxinA; 154 units/ng for abobotulinumtoxinA; and 227 units/ng for incobotulinumtoxinA [112]. A summary of the literature about safety and maximum doses is reported in Table 1.

The first reported case of MMH treatment, described by Moore and Woode in 1994 [14], used 300 units of abobotulinumtoxinA in a 30-year-old man with TMJ dysfunction, myofascial pain syndrome, and masseter hypertrophy. The patient exhibited a slight muscle reduction for two weeks post-injection, with effects lasting six months without side effects. One of the largest studies, conducted by Kim et al. [70], involved 1021 patients treated with 100–140 units of abobotulinumtoxin A (ABO) for lower face contouring (383 patients could be followed for >3 months). The thickness was reduced by 31% and the maximum reduction in muscle strength was observed after 10–12 weeks and 50% required a second injection after 4–7 months. Then, the large-scale trial by Kim et al. [70] reported consistent success using lower doses, indicating that modern approaches are increasingly effective with reduced toxin volumes. A reduction in symptoms can be achieved with doses less than 25U applied exclusively to the masseter muscles [97]. However, recommendations most commonly suggest using a total amount of 24 to 60 U of onabotulinumtoxinA or incobotulinumtoxinA and 60 to 300 U of abobotulinumtoxinA at three sites, with a 30 G needle inserted deep into the muscle [117]. A higher bilateral dose of 60–100 U could instead be the optimal choice for the treatment of muscle TMD pain. Notably, a higher dose of BoNT/A resulted in greater pain reduction at 6 months compared to a lower dose [102].

## 8. Side Effects, Complications, and Contraindications of Botulinum Toxin for Masseter Muscle Hypertrophy

### 8.1. Side Effects of Botulinum Toxin for Masseter Muscle Hypertrophy

Botulinum toxin injections into the masseter muscle are widely regarded as safe and effective for both therapeutic and aesthetic applications. BoNT/A has proven to be a safe treatment, even after repeated sessions for several years [118]. All studies focus on identifying a safe injection area rather than on the specifics of the technique [111]. There were favorable results by selecting the locations and depth of BoNT/A applications by clinical estimation and palpation, with few or no reports of adverse effects [97]. The safe injection area is usually delimited by the lower mandibular border, the margins of the anterior and posterior masseter, and the zygomatic arch as the upper limit. Various side effects (intended as predictable, generally mild, and often dose-dependent outcomes of treatment) associated with the procedure have been reported, most likely secondary to excessive infiltration or inaccurate placement of the BoNT/A, albeit up to 50% of patients may experience adverse events (as harmful and unintended outcomes, which may range from mild to severe and may not always be directly linked to the treatment), which are mostly mild, transient, and self-resolving within 1–2 months [119]. Table 2 depicts the main side effects of BoNT/A for MMH management [51,71,77,82,107,118,120,121,122,123].

The most common complications of injections (as unintended and often preventable deviations from the expected course of treatment) include mild pain at injection sites or temporary muscle weakness (e.g., atonia of the injected muscle), which can lead to unwanted weakness or functional limitations such as reduced chewing strength and difficulty opening the mouth [124]. Aesthetic concerns include asymmetry of the smile—particularly in facial applications—problems opening the mouth, bruising (accounting for 2.5%), incipient sagging of the cheeks and skin, local pain at the injection site, mild headache, awkward facial expressions, speech disorders, irregular swelling of the muscles, sunken cheeks, and herniation of the parotid gland through the overlying attenuated muscle. After treatment, due to the rapid reduction in size of the masseter muscle, sagging of the skin and soft tissue in the jaw area may occur in 0.2% to 2.3% of patients, resulting in a blurred and more aged appearance of the jaw [75]. These are particularly common in older patients or those with thinner skin as rapid masseter muscle atrophy can lead to skin laxity of the soft tissues of the mandibular border and a more aged appearance [14]. This is mainly caused by gravity and the tensile force of the platysma muscle [72]. Treatment strategies to mitigate these side effects include lower doses and spaced sessions as well as complementary injections into depressor muscles to counteract sagging [65]. These effects are often dose-dependent, localized, and uncommon [107] and can be minimized by employing precise injection techniques and tailoring the dosage to individual patient anatomy and needs [51,125]. Furthermore, since the effect of BoNT/A is reversible, muscle weakness gradually recovers over the course [84]. Functional side effects include transient facial asymmetry and altered chewing mechanics, which can result from unintended weakening of adjacent muscles such as the risorius or zygomaticus. Two patients in the study by Kim et al. [70] reported aesthetic changes in their smile, which may be due to the diffusion of botulinum toxin toward the zygomaticus and risoris muscles during application. To avoid this, the BoNT/A must be injected well within the confines of the masseter muscle and, superiorly, a line joining the tragus to the oral commissure must be considered as the superior margin.

### 8.2. Complications of Botulinum Toxin for Masseter Muscle Hypertrophy

BoNT-A is widely regarded as a safe and effective therapeutic agent, yet rare complications, though infrequent, demand attention and careful management. Occasionally, paradoxical muscle swelling may occur, presenting as localized bulging despite the intended reduction in muscle activity. This phenomenon is often linked to uneven toxin diffusion or anatomical barriers, such as the deep inferior tendon within the masseter muscle. Ultrasound guidance and carefully distributed injections are key to preventing such outcomes [126,127]. Other rare effects include dysphagia and speech difficulties resulting from unintended spread to the adjacent muscles, as well as transient facial nerve palsy causing asymmetry or weakness. These complications can be avoided with refined injection techniques and precise localization of the toxin [62]. Systemic toxicity, while extremely rare, underscores the importance of adhering to safe dosing practices and immediate intervention in case of overdose [119]. Long-term use of BoNT/A in masticatory muscles has raised concerns about mandibular bone thinning, particularly in animal studies. Several studies have examined the impact of BoNT/A injections on mandibular bone structure [123,124,125,126,127]. Moussa et al. [123] have recently reported, in a systematic review and meta-analysis, the adverse effects of BoNT/A injections on mandibular bone tissue. The authors reviewed both preclinical and clinical studies to assess the impact of BoNT/A dosage on bone density and mandibular structure. We recognize that the dose–response relationship is a critical factor in interpreting the findings of both preclinical and clinical studies. The author found that in animal studies, higher doses of BoNT/A were employed relative to muscle mass compared to the doses used in human studies. Conversely, human studies typically used doses within the therapeutic range (e.g., 20–50 units per side for masseter hypertrophy), which are less likely to result in significant bone changes. Therefore, the higher doses in animal models have demonstrated more pronounced effects on mandibular bone density and microarchitecture, while lower, therapeutic doses in humans appear to have minimal or no clinically significant impact. However, the authors concluded that while the preclinical evidence points to negative effects of BoNT/A on mandibular bone health, the clinical evidence remains insufficient to draw definitive conclusions [123]. They recommend caution with prolonged or high-dose BoNT/A use in masticatory muscles and emphasize the need for further clinical research to assess the long-term impact of these treatments on mandibular bone structure. Hong et al. [124] observed a reduction in cortical bone quality following BoNT/A injections, highlighting the potential for bone structure deterioration due to decreased mechanical loading. Research by Tsai et al. on rats [125] indicated that BoNT/A injections into masticatory muscles led to reduced cortical bone thickness in specific mandibular regions, such as the angular process, due to muscle atrophy and subsequently decreased mechanical forces. Kahn et al. [126] reported that BoNT/A-induced muscle atrophy resulted in decreased bone quality in the mandibular condyle and alveolar bones, emphasizing the relationship between muscle function and bone integrity. These studies collectively suggest that BoNT/A injections can lead to a reduction in mandibular bone quality, particularly in cortical thickness, due to the interruption of normal bone remodeling processes. The regions of interest commonly affected include the mandibular condyle, coronoid process, and angle/ramus, with significant changes observed within 3 to 12 months post-injection [126]. Therefore, mandibular bone loss could be considered an adverse effect of BoNT/A’s application on masticatory muscles; even though more studies are needed, the results extrapolated to humans [102] highlight the need for caution in prolonged or high-dose treatments [126]. Allergic reactions such as allergic dermatitis or antibody formation, although rare, have been described in a small percentage of patients, while systemic reactions of flu-like symptoms or generalized malaise are exceedingly rare [127]. These reactions are usually mild and self-limiting [127]. Genetic predispositions and immune variability among patients may further influence susceptibility to these events [120]. Management strategies focus on using the lowest effective dose, extending intervals between treatments, and considering alternative formulations when antibody resistance is suspected [120]. Huang et al. [66] demonstrated that three-dimensional photography combined with ultrasound could track volume reduction more accurately, enabling better patient satisfaction and tailored treatments. The injection of botulinum toxin into the orofacial region can also be guided with the help of electromyography [110,126]. To reduce the incidence of rare complications associated with BoNT/A treatments, practitioners can employ several preventative strategies focusing on precision, technique, and patient-specific factors, including the use of ultrasound-guided injections.

To mitigate immune-mediated responses, it is crucial to minimize cumulative doses and extend the intervals between treatments. For patients who develop resistance due to neutralizing antibody formation, switching to alternative toxin formulations may restore efficacy [121]. Finally, patient education cannot be overlooked. Informing patients about potential transient effects, such as mild chewing weakness or temporary facial asymmetry, prepares them for recovery. Advising rest post-treatment can further help reduce minor side effects like dizziness or headaches. With a combination of advanced guidance tools, careful injection planning, personalized treatment approaches, and patient education, clinicians can significantly reduce the risk of rare complications. These strategies ensure safer and more effective outcomes in botulinum toxin therapies [69,93,118,124,127]. Table 3 depicts the risks and mitigation methods in detail.

## 9. Limitations of This Study

The present study has the main limitation of being a narrative review. Thus, this study’s design did not allow us to provide evidence according to systematic research of the scientific literature, nor did it provide quantitative conclusions. Additionally, variability in the designs, sample sizes, and methodologies across the included studies is a limiting factor. Furthermore, the lack of randomized controlled trials comparing botulinum toxin protocols for masseter hypertrophy represents another limitation.

## 10. Conclusions

In summary, BoNT/A injections in the masseter muscle are a valuable tool for both therapeutic and aesthetic purposes. The treatment of MMH with botulinum toxin requires a careful balance of dosage, technique, and frequency to achieve both functional and aesthetic improvements. While significant advancements have been made in refining protocols, the variability in each patient’s anatomy and needs underscores the importance of individualized treatment planning. BoNT-A is effective and safe in the treatment of MMH and TMDs, offering significant benefits in terms of pain reduction, improvement in muscle and joint function, and aesthetic advantages, with effects lasting several months. While most side effects are mild and self-limiting, rare complications underscore the importance of precision, advanced techniques, and individualized treatment planning. By utilizing innovations like ultrasound-guided injections, clinicians can further enhance the safety and efficacy of these treatments. Continued research into the long-term effects of BoNT/A, particularly on mandibular bone health, is essential to ensure optimal patient outcomes. Future studies are needed to establish more definitive guidelines for optimal dosing, injection sites, and treatment intervals and to better understand the mechanisms underlying botulinum toxin’s effects on muscle function.

## Figures and Tables

**Table 1:** Injection techniques and doses for masseter muscle hypertrophy.

Injection Technique	Number of Injection Sites	Total Dose	Toxin Type	Notes (with References)
Multi-point injection	3–5	24–60 U (onabotulinum/incobotulinum toxin A)	Onabotulinum/Incobotulinum	Ensures uniform toxin distribution within the muscle and reduces asymmetry risks [91,112]
Single-point injection	1	20–40 U (onabotulinum toxin A)	Onabotulinum	Faster and less painful; may result in uneven muscle reduction [108,113,114]
Ultrasound-guided injection	Variable	20–60 U	Onabotulinum/Incobotulinum	Increases precision, reduces diffusion risks, and ensures accurate delivery [62,91,108]
Multi-muscle protocol (masseter, temporalis, and in some cases, the pterygoid muscles)	Variable	20–60 U (masseter, temporalis, pterygoid)	Onabotulinum	Associated with significant pain reduction in TMD patients [71]
Abobotulinum toxin A	3	60–300 U	Abobotulinum	BoNT-A neurotoxin is estimated at 137 units/ng for onabotulinumtoxinA, 154 units/ng for abobotulinumtoxinA, and 227 units/ng for incobotulinumtoxinA [112,113,114]
High-dose injection	Variable	Up to 400 U every 3 months	All types	Effective for severe TMD; no significant increase in adverse events with higher doses [14,115]
Repeat treatment	Variable	15–30 U per side	Onabotulinum	Muscle thickness and dosage decrease with repeated injections over time [116]
Combination therapy (masseter + others)	3+	60–100 U (masseter, temporalis, pterygoid)	Onabotulinum	Combined improvement in TMD symptoms and facial contouring [71,92]

**Table 2:** Side effects of botulinum toxin treatment for masseter muscle hypertrophy.

Incidence	Side Effects	Management
Common (~30–50%)	Mild pain at injection site, temporary muscle weakness	Analgesics, reassurance; dose adjustment
Occasional (~2–15%)	Facial asymmetry, bruising, headache, altered chewing mechanics, sunken cheeks	Precise injection techniques; local compression; analgesics; tailor dosage to individual anatomy
Rare (~0.2–2.3%)	Incipient sagging of cheeks/skin, sagging of jaw soft tissues	Use lower doses; distribute sessions; consider depressor muscle injections
Rare (~<1–2%)	Aesthetic changes in smile, irregular swelling of muscles, speech disorders, herniation of parotid gland, bone remodeling changes	Inject within masseter boundaries; employ ultrasound guidance; monitor and adjust treatment
Very Rare (<1%)	Immune-mediated reactions	Cease treatment; consult immunologist

**Table 3:** Risks and mitigation methods for botulinum toxin treatment.

Risk	Mitigation Method
Facial asymmetry	Ensure proper pre-treatment assessment and dose adjustments; inject within safety zones.
Chewing weakness	Use the lowest effective dose; avoid excessive infiltration; use ultrasound or EMG guidance.
Bruising at injection site	Apply local compression immediately post-injection; use smaller-gauge needles.
Sagging skin and soft tissues	Lower injection doses; distribute treatment over several sessions; complementary injections in depressor muscles.
Paradoxical swelling	Use ultrasound guidance to confirm masseter anatomy; superficial booster doses may help if swelling persists.
Diffusion to unintended muscles (e.g., risorius)	Ask patients to clench their teeth during injection to identify muscle boundaries; inject well within masseter confines.
Bone changes (e.g., osteopenia)	Avoid prolonged or high-dose treatments; monitor long-term effects; further research needed for validation.
Unnatural facial expressions	Inject at appropriate depth and within masseter boundaries; adjust doses based on patient anatomy.
Pain at injection site	Use precise injection techniques and ensure proper patient positioning.

Legend: This table outlines the key risks associated with botulinum toxin treatment and the corresponding mitigation methods. The risks range from mild, transient effects like bruising and pain at the injection site to more significant complications such as facial asymmetry, sagging skin, or unintended diffusion of the toxin. Mitigation strategies include precise injection techniques, ultrasound guidance, dose adjustments, and appropriate pre-treatment assessments to ensure patient safety and optimal therapeutic outcomes.

## Data Availability

No new data were created or analyzed in this study. Data sharing is not applicable to this article.
